# Personalized Smart Home Automation Using Machine Learning: Predicting User Activities

**DOI:** 10.3390/s25196082

**Published:** 2025-10-02

**Authors:** Mark M. Gad, Walaa Gad, Tamer Abdelkader, Kshirasagar Naik

**Affiliations:** 1 Media Engineering and Technology (MET) Department, German University in Cairo, Cairo 11835, Egypt; magedmark50@gmail.com; 2 Faculty of Computer and Information Sciences, Ain Shams University, Cairo 11566, Egypt; walaagad@cis.asu.edu.eg; 3 Faculty of Computer Science and Engineering, Galala University, Suez 435611, Egypt; tamer.abdelkader@gu.edu.eg; 4 Department of Electrical and Computer Engineering, University of Waterloo, Waterloo, ON N2L 3G1, Canada

**Keywords:** smart home automation, machine learning, human activity recognition, edge computing, intelligent environments, gradient boosting models, personalization, context-aware systems

## Abstract

A personalized framework for smart home automation is introduced, utilizing machine learning to predict user activities and allow for the context-aware control of living spaces. Predicting user activities, such as ‘Watch_TV’, ‘Sleep’, ‘Work_On_Computer’, and ‘Cook_Dinner’, is essential for improving occupant comfort, optimizing energy consumption, and offering proactive support in smart home settings. The Edge Light Human Activity Recognition Predictor, or EL-HARP, is the main prediction model used in this framework to predict user behavior. The system combines open-source software for real-time sensing, facial recognition, and appliance control with affordable hardware, including the Raspberry Pi 5, ESP32-CAM, Tuya smart switches, NFC (Near Field Communication), and ultrasonic sensors. In order to predict daily user activities, three gradient-boosting models—XGBoost, CatBoost, and LightGBM (Gradient Boosting Models)—are trained for each household using engineered features and past behaviour patterns. Using extended temporal features, LightGBM in particular achieves strong predictive performance within EL-HARP. The framework is optimized for edge deployment with efficient training, regularization, and class imbalance handling. A fully functional prototype demonstrates real-time performance and adaptability to individual behavior patterns. This work contributes a scalable, privacy-preserving, and user-centric approach to intelligent home automation.

## 1. Introduction

Smart home automation uses sensor networks and signal processing to create adaptive environments that engage with human behavior intelligently. As a means of improving user comfort, efficiency, and safety, modern systems do not just aim at automating routine activities but also to learn and predict the user’s behavior proactively. Machine learning-based approaches are increasingly being utilized within domestic technology to monitor, anticipate, and react to everyday human activity in real time. This allows for more discriminating energy management, security, and care-at-home services.

Conventional automation platforms are often limited by static rules, fixed schedules, or user-programmed scenes. These systems are useful in straightforward situations; however, they are not adaptable enough to handle behavioral variability and customization. Furthermore, system design and data processing become even more complex when heterogeneous devices—from cameras and motion sensors to smart switches and voice assistants—are integrated. These difficulties call for learning-based systems that can recognize patterns in behavior and make adjustments on their own.

In smart environments, machine learning—especially supervised learning—has become a popular method for predicting and identifying activity. Even though deep learning models—like convolutional neural networks (CNNs) and long short-term memory (LSTM) networks—have shown encouraging results, they frequently call for large amounts of labeled data and significant processing power. Furthermore, deep neural networks’ inability to be interpreted poses problems for applications that must be safe and user-facing. In contrast, gradient boosting decision tree (GBDT) ensembles—such as XGBoost, CatBoost, and LightGBM—offer strong predictive performance, interpretable outputs [1], and efficient training on structured tabular data. These characteristics make GBDT models particularly suitable for smart home sensor data, where time-series information is often presented in fixed-length sequences and categorical formats [2].

Enhancing prediction accuracy necessitates time-aware feature engineering. Without the need for intricate recurrent architectures, models can learn user routines through temporal indicators such as time of day, day of the week, rolling activity statistics, and historical trends. Furthermore, it has been demonstrated that user-level personalization, in which distinct models are trained for each resident, enhances generalization and system responsiveness.

The main goal of this work is to build a system that is able to predict each unique user’s future behavior to enable personalized automation in residential settings. This system can proactively automate devices, optimize energy consumption, and enhance comfort and safety by precisely predicting human activities, such as cooking, sleeping, or leaving the house. The ability to precisely predict human activities, such as leaving the home or going to sleep, can directly enhance energy management beyond simple automation. By anticipating future behavior, the system can proactively limit the use of unnecessary appliances, optimize climate control schedules, and cut off any unused power to devices that are about to become idle. This proactive approach ensures that resources are not wasted, leading to significant reductions in overall power consumption and contributing to a more sustainable and cost-effective smart home environment. Instead of reacting to static schedules or direct user input, the suggested framework learns from past activity patterns and contextual cues to predict needs and carry out control decisions automatically. The core prediction model EL-HARP is deployed within this framework to perform these activity forecasts. This predictive capability allows a seamless orchestration of appliances, lighting, and environmental controls in a way that adapts to each resident’s habits and lifestyle.

In addition to developing a machine learning-based prediction pipeline, this work also aims to introduce a functional smart home system design that demonstrates a practical method for collecting behavioral data in real residential settings. The CASAS dataset [3] is employed for model training and evaluation; however, the system architecture includes physical sensor deployments such as facial recognition modules, NFC tags, ultrasonic presence sensors, and smart switches—to emulate realistic residential scenarios. These components collectively form a prototype environment capable of recording, labeling, and responding to user activities, thereby laying the foundation for future datasets based on real-time, in-home deployments.

During the development of the proposed smart home activity prediction framework, several key challenges were encountered:

1. Sensor noise and class imbalance in the CASAS dataset affected learning reliability. Several activity classes exhibited sparse and inconsistent representation, while others included overlapping or ambiguous sensor patterns. Labels such as “Other Activity” and “Entertain Guests” were found to be frequent sources of noise and were excluded through targeted preprocessing [4].
2. Imbalanced class distributions can lead to high overall accuracy but poor accuracy for underrepresented classes. This discrepancy is particularly evident when comparing accuracy with the weighted F1-score, revealing skewed recognition performance across different activity types.
3. Computational constraints prevented the use of many state-of-the-art deep learning architectures. Real-time deployment on edge hardware such as the Raspberry Pi 5 requires lightweight and interpretable models, necessitating trade-offs between model complexity and performance [2].
4. Label ambiguity and activity overlap have also been recognized as significant challenges during model training. Sensor events triggered by activities such as “Relaxing” and “Watching TV” often exhibited high similarity, hindering clear class separation. These issues have been previously reported in the literature [4], and their effects were observed during both data preprocessing and evaluation.

To address these challenges, the EL-HARP framework was designed as a modular, edge-deployable system for personalized activity prediction and automation. Raw sensor streams are first ingested via a temporal feature-extraction pipeline that denoises events, computes instantaneous, rolling, and historical embeddings, and assembles fixed-length input sequences. These sequences are then fed into per-user gradient-boosted tree ensembles (XGBoost, CatBoost, LightGBM) to produce real-time activity forecasts. EL-HARP orchestrates all components data preprocessing, on-device inference, automation logic, and interactive labeling within Docker containers running on a Raspberry Pi 5. Performance was measured in terms of prediction accuracy, weighted F1-score, and inference latency on the edge. A fully functional prototype demonstrated EL-HARP’s ability to generalize across heterogeneous sensor inputs, maintain sub-100 ms response times, and continuously adapt to user behavior via incremental, voice-driven retraining.

The primary contributions of this work are summarized as follows:

- A comprehensive, modular smart home automation framework is proposed, integrating real-time sensing and actuation with personalized prediction of a wide range of user activities, including Watch_TV, Sleep, Leave_Home, Cook_Dinner, and Personal_Hygiene.
- A novel time-aware feature engineering strategy is developed, combining temporal signals and historical behavior patterns to enhance the accuracy and interpretability of activity prediction models. This strategy, applied to gradient-boosting models (XGBoost, CatBoost, LightGBM), achieves particularly strong predictive performance with LightGBM.
- The proposed framework and prediction approach demonstrate practical and efficient deployment on constrained edge devices, enabling a privacy-preserving and adaptive smart home solution designed for real-world environments.

The remainder of this paper is organized as follows: Section 2 provides an overview of related work in smart home automation and activity prediction. Section 3 details the proposed EL-HARP framework, including its architecture, hardware components, and software integrations. Section 4 elaborates on the data-processing pipeline, feature engineering strategies, and the machine learning models employed for activity prediction. Section 5 presents the experimental setup, discusses the evaluation methodology, and analyzes the performance results. Finally, Section 6 concludes the paper with a summary of key findings and outlines directions for future work.

## 2. Related Work

Smart home automation has advanced significantly recently, leveraging development in machine learning techniques and high-performance edge computing platforms and continuously developing human activity recognition (HAR) datasets to analyze and predict the user activities and optimize resource utilization. This section will go through recent contributions across HAR datasets, edge computing platforms, and machine learning techniques used in this field.

### 2.1. Datasets for Smart Home Automation

Annotated, high-quality datasets are pivotal for human activity recognition research. The following are recent notable datasets that are frequently used.

- MuRAL [5]: MuRAL, the a multi-resident ambient sensor dataset with natural language annotations, was first made available in 2025. It consists of more than 21 h of multi-user sensor data gathered from 21 smart-home sessions. Research on multi-resident activity recognition and natural language comprehension is facilitated via the dataset’s inclusion of resident identities, high-level activity labels, and fine-grained natural language descriptions. However, since it is a newly released dataset, it lacks established benchmarks in the literature and has seen limited adoption in studies, which may pose challenges for comparative evaluation and generalizability.
- CASAS-SMART consists of a sizable, long-term collection of ambient sensor data from actual homes and is a popular public resource for human activity recognition (HAR) research. Time-stamped events from basic, non-intrusive sensors, such as motion and door sensors, which are discreetly installed to monitor daily activities, are the main source of data for the dataset. Researchers can train and assess machine learning models to infer human actions from sensor patterns by labeling this raw data stream with particular activities. The dataset is not only used for creating predictive HAR models but also for creating anomaly detection and ambient assisted living systems, where spotting departures from typical behavior can be crucial for keeping an eye on wellbeing and health.
- Smart Meter Dataset [6] includes power readings for several different households. It is employed in hybrid transformer–RNN architectures that prioritize highly accurate and privacy-aware activity forecasting and recognition.
- Opportunity Dataset is employed to identify human activity through the use of ambient and wearable sensors. It contains sensor-rich recordings with numerous annotated activities, which are frequently used for sequence modeling in deep learning research.

### 2.2. Edge Computing Platforms

Deploying machine learning models within home environments necessitates efficient, low-power hardware solutions, such as the following.

- Home Assistant Appliances [7]: Zigbee, Thread, and Matter protocols are supported via devices such as Home Assistant Yellow and Green, which provide integrated solutions. Although they support a wide range of protocols and are easy to use, their onboard AI processing power is limited.
- NVIDIA Jetson Platforms [8]: For deep learning tasks, offer GPU acceleration that is appropriate for computationally demanding applications. Although they are more expensive and use more power, they provide excellent AI performance and scalability.
- Raspberry Pi (RPi) Systems [2] are used as direct-sensor central controllers that facilitate data storage, automation, and remote control at the network edge. Although they are flexible and reasonably priced, their processing power is constrained for intricate models.

### 2.3. Machine Learning for Energy Management and Optimization

Beyond activity recognition, machine learning is a cornerstone for optimizing power consumption and integrating renewable energy sources in smart homes. This field leverages predictive and control-based methods to create more efficient and sustainable systems.

#### 2.3.1. Prediction of Power Generation

To effectively manage energy from renewable sources, the accurate forecasting of power generation is essential. Recurrent Neural Networks (RNNs) and their advanced variants, such as Long Short-Term Memory (LSTM) networks, are particularly well suited to this task. These models excel at processing and learning from sequential data, making them ideal for predicting solar power output based on historical time-series data of weather, temperature, and cloud cover. By providing precise forecasts, these models enable energy management systems to make informed decisions about when to store energy, consume it, or sell it back to the grid.

#### 2.3.2. Reinforcement Learning for Control

For dynamic energy management, Reinforcement Learning (RL) and Deep Reinforcement Learning (DRL) offer a powerful framework. In this approach, an intelligent agent learns to make optimal control decisions by interacting with the smart home environment to maximize a long-term reward, such as minimizing energy costs or enhancing occupant comfort. Research in this area includes using RL for modulating specific systems like heat pumps and photovoltaic systems [9] and for managing demand response using historical data [10]. More advanced models, like multi-agent reinforcement learning, are also being explored [11].

- Optimal Power Scheduling: DRL has been used to create automated systems that manage demand response. For instance, in “An optimal power scheduling method for demand response in home energy management system” [12], a system learns to shift the operation of appliances to off-peak hours to reduce electricity bills.
- Autonomous Control: More advanced methods, such as Actor-Critic learning, enable agents to manage complex systems like HVAC and battery storage simultaneously. The paper “Autonomous Price-aware Energy Management System in Smart Homes via Actor-Critic Learning with Predictive Capabilities” [13] proposes a system that uses DRL to make real-time decisions based on electricity prices and predicted energy needs, balancing cost and comfort.
- Electric Vehicle (EV) Charging: The optimization of EV charging is a critical application. Researchers have used DRL to develop “effective charging planning” [14] strategies that minimize charging time. Similarly, a Continuous Deep Deterministic Policy Gradient (CDDPG)-based approach has been introduced for precise and continuous control of EV charging to manage grid load and reduce costs [15].

These applications highlight the shift from reactive to proactive and predictive energy management, positioning machine learning as a core component of future smart home systems.

### 2.4. Machine Learning Techniques

Recent developments in smart-home human activity recognition use a wide range of machine learning techniques, from edge-optimized architectures and techniques robust to incomplete data to self-supervised learning approaches. Every one of these approaches offers advantages and disadvantages that affect accuracy, resource usage, and deployment suitability. A selection of important studies is highlighted below. Non-intrusive techniques using ambient sensors like Wi-Fi signals and cameras are gaining prominence [16,17].

Additionally, new sensing techniques are always being investigated; for instance, thermal imaging combined with Internet of Things devices has proven useful for identifying activity in homes [18]. In a related study, Lin et al. (2023) [19] explored a suite of machine learning algorithms, including gradient boosting, to infer user activities directly from heterogeneous IoT device network traffic, demonstrating the viability of a non-intrusive and privacy-preserving approach based on network flow patterns.

Ali et al. (2025) [20] introduced an Innovative IoT and Edge Intelligence Framework for monitoring senior citizens by employing anomaly detection from sensor data from non-wearable devices. This system, which was created especially for edge deployment, is a good contender for real-time home health monitoring because it obtained 82.36% Precision and 86.03% F1 Score on the CASAS TM029 dataset.

Feng et al. (2024) [21] suggested a centralised Intensive Care Unit (ICU) Command Centre Architecture that can use Transformer models and attention mechanisms to fuse medical data, including smart home sensors. The suggested masked modeling technique can be applied to HAR settings where missing sensor events are common, even though the original study concentrated on critical care applications. Their architecture achieved F1 scores of 85.0% on the CASAS Aruba and 64.0% on the CASAS Milan datasets, demonstrating strong robustness to incomplete inputs.

Chen et al. (2024) [22] incorporated Self-Supervised Learning with Self-Attention, efficiently utilizing unlabeled data to lessen reliance on manual annotation. Their method demonstrated strength in pretraining but limitations under minimally labeled conditions, achieving 85.63% F1 (Aruba-1) and 59.74% (Milan).

Srivatsa & Plötz (2024) [23] employed Graph Neural Networks to simulate the interactions of smart home sensors. The technique records intricate spatial–temporal patterns by converting sensor events into graph representations. Across several CASAS datasets, F1 scores varied from 78.3% to 88.7%.

Fiori et al. (2025) [24] presented GNN-XAR (Graph Neural Network-Explainable Activity Recognition), an explainable GNN framework that is tailored for HAR. With an average accuracy of 86.5% on the CASAS Milan and Aruba datasets, it highlights model transparency through the use of attention-based graph modelling.

Zhou et al. (2022) [25] created TinyHAR, a small deep learning model designed for deployment on the edge. Its practical utility is demonstrated by the fact that it maintains high performance up to 89.0% accuracy across various HAR datasets while reducing the model size by >90% when compared to baselines.

Khan et al. (2022) [26] put into practice a Hybrid Deep Learning Model for HAR in smart homes that combines CNN and Bi-LSTM layers. Both temporal and spatial features are successfully captured through the dual architecture. Despite its strength, it requires more computing power than non-deep models. On the CASAS dataset, its accuracy was 89.0%.

Khan et al. (2025) [27] introduced a Multimodal Temporal Transformer for HAR, which focuses on fusing features from diverse sensors to capture complex temporal dependencies. This deep learning approach offers a powerful alternative to traditional methods but is computationally expensive and less suited for real-time edge deployment.

Dao et al. (2025) [28] proposed RFAR, a real-time system for firefighter activity recognition using wearable accelerometers. While achieving a very high accuracy of 97.35% on the UCI HAR dataset, this approach is limited to a specific application and relies on wearable, single-modality sensors, which differs from the multi-sensor ambient environment of smart homes.

Yang et al. (2025) [29] presented a privacy-preserving HAR method by fusing Inertial and High-resolution Acoustic Data. This work highlights the importance of data fusion and privacy, though its deep learning fusion model is more complex than a GBDT approach.

Li et al. (2023) [30] developed an approach for HAR based on Multi-environment Sensor Data. Their deep learning model, HENN-MSD, achieved a state-of-the-art accuracy of 96.57% on the CASAS dataset, showcasing the performance potential of complex deep architectures for activity generalization.

Furthermore, deep unified models combining convolutional neural networks with edge computing have been applied for tasks like face recognition [31], while ensemble boosting methods like XGBoost have been shown to improve the consistency of accuracy in various classification tasks [32]. More recent work explores few-shot learning with MLLMs and visual reinforcement learning to advance HAR [33]. An overview is given in Table 1.

### 2.5. Open Challenges

Despite significant advancements, several challenges persist, as follows.

- Personalization vs. Privacy: Balancing model accuracy with user data privacy, especially in multi-resident scenarios. The use of tailored small language models on edge devices is a promising approach to this issue [34], as is the use of machine learning to detect cyber attacks [35]. Generative AI could also be used to simplify the user-centric setup of these systems [36].
- Generalization: Ensuring models perform well across diverse home environments and sensor configurations. This includes the challenge of continuous adaptation and continual learning to avoid forgetting past knowledge [37].
- Edge Constraints: developing models that operate efficiently on resource-constrained edge devices.
- Robustness: handling sensor noise, missing data, and unpredictable user behaviors.

Future studies should concentrate on creating interpretable, lightweight models that can learn customized routines while protecting user privacy and performing well on edge devices. In this regard, EL-HARP is presented as a portable, interpretable model created especially for effective, private activity prediction in smart home settings with limited resources.

## 3. Methodology

This section outlines EL-HARP, a unified framework for personalized activity recognition and automation in smart homes. It integrates a real-world IoT prototype with on-device machine learning tailored for edge deployment. EL-HARP encompasses the full sensing-to-action pipeline—real-time data collection, temporal feature extraction, and a lightweight, adaptive gradient-boosted ensemble—running entirely on embedded hardware. The system refines its behavior through incremental learning and user feedback, ensuring continuous personalization and responsiveness without relying on cloud services.

The end-to-end framework comprises the following components:

1. A physical smart home prototype integrating commodity sensors and embedded controllers;
2. Five real-time functional blocks supporting live activity detection and automation;
3. A structured logging subsystem for collecting appliance and behavior patterns;
4. A full data processing pipeline encompassing filtering, feature extraction, and temporal encoding;
5. Gradient boosting–based activity classification and continuous retraining at the edge.

The system is designed to operate entirely offline, support user-specific routines, and evolve over time based on in-home feedback. All inference and learning are performed on-device, preserving privacy and enabling personalized automation without relying on centralized servers.

**Figure 1:**
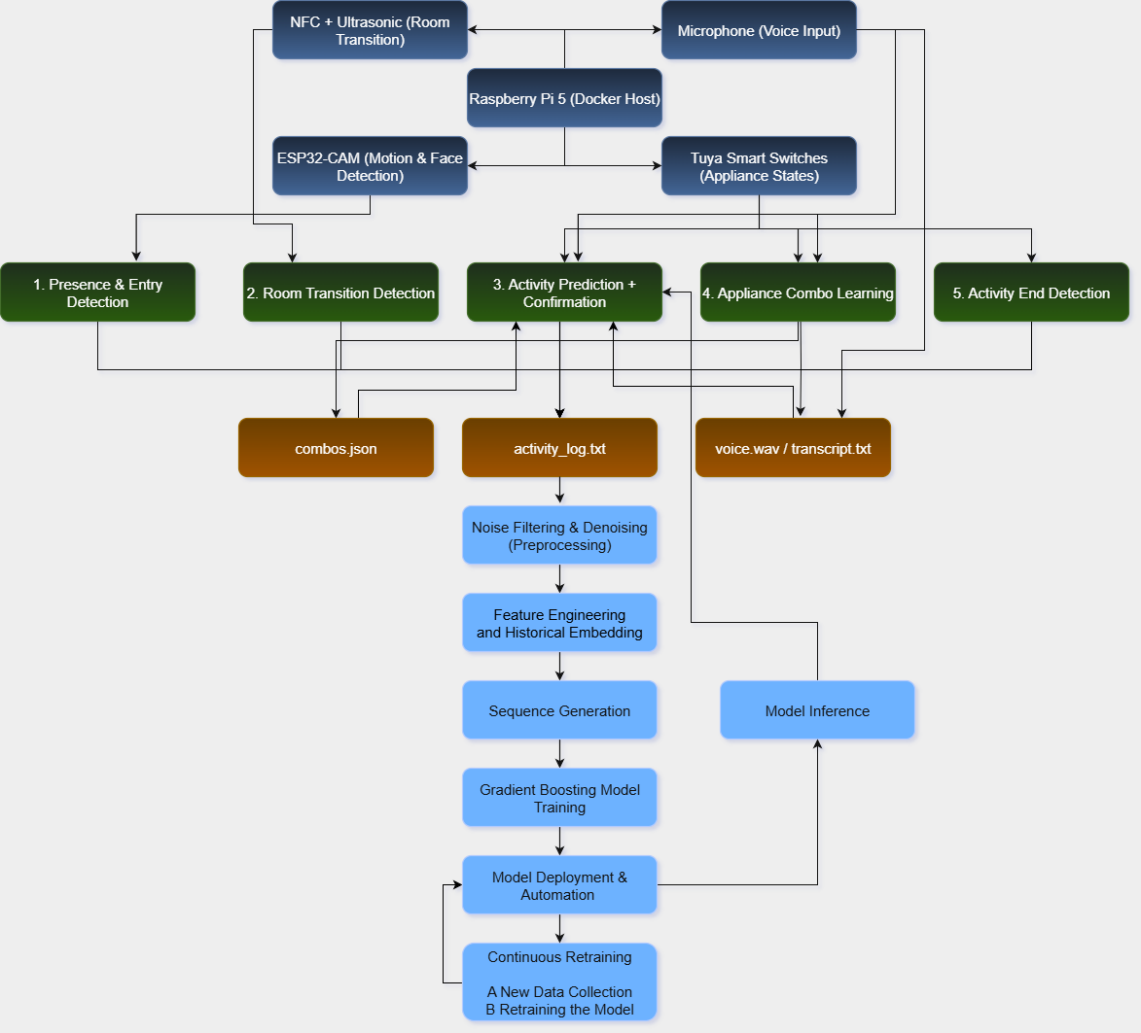
System architecture of the EL-HARP framework, showing the hardware interface, five real-time control functions, structured logging, and the downstream learning pipeline.

Figure 1 provides a visual summary of this architecture. The top section shows the physical sensing modules connected to a Raspberry Pi 5 controller, while the middle layer outlines the five main functional components of the system. These include presence detection, room transition handling, activity prediction with confirmation, combo-based learning, and activity end detection.

Outputs from these components are written to structured files (activity_log.txt, combos.json, and voice transcripts), which feed into a multi-stage processing pipeline. The data undergoes filtering, feature engineering, and sequence generation before being used to retrain the underlying machine learning model. The bottom segment of the diagram highlights the continuous loop: data collected from live operation directly informs model updates and redeployment—closing the loop between sensing, inference, and learning.

Together, these components validate that personalized smart home intelligence can be realized with embedded, privacy-preserving systems that evolve over time without central cloud reliance.

### 3.1. Hardware and System Architecture

The smart home prototype is built around a Raspberry Pi 5 (8 GB) that hosts all core services in Docker containers. Five types of sensors and devices connect to the Pi to capture user presence, location, and appliance usage:

- ESP32-CAM Modules: Mounted at each entrance, they provide motion-triggered video streaming. Video frames are forwarded to the Pi for OpenCV-based face detection and recognition.
- NFC Readers + Ultrasonic Sensors: Installed at door thresholds to detect room-to-room transitions. NFC tags carried by residents identify the user, while the ultrasonic sensor confirms directional movement.
- Tuya-Compatible Smart Switches: Deployed on major appliances (lights, TV, kettle). Their on/off states are polled via Home Assistant to infer ongoing activities.
- Microphone: Captures short voice responses during user confirmation or when labeling unknown appliance combinations. Audio is stored temporarily and passed to a local Whisper engine.
- Raspberry Pi 5: Serves as the central controller, running:
  - OpenCV Container: For face recognition.
  - Home Assistant Container: For device polling and state management.
  - Node-RED Container: For orchestrating logic flows.
  - Whisper Container: For offline voice transcription.
  - Inference Engine Container: Hosting the EL-HARP LightGBM model.

**Figure 2:**
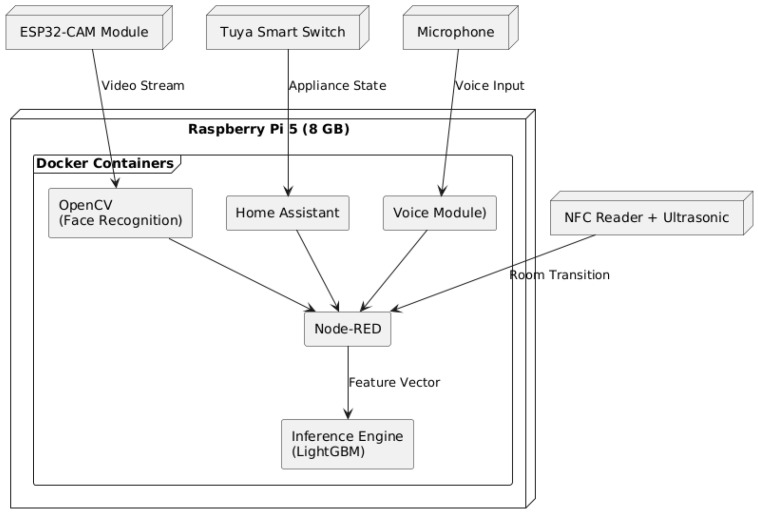
Hardware and system architecture of the smart home prototype: sensing modules feed into Dockerized services on Raspberry Pi 5, which in turn manage control logic, logging, and model inference.

All devices communicate locally over the home network; no data is transmitted externally. Figure 2 illustrates this layered architecture.

### 3.2. Real-Time Functional Blocks

Five real-time subsystems run on the Raspberry Pi within Node-RED to detect presence, track location, predict activities, learn new appliance combinations, and detect activity end. All subsystems log events in the format:

Timestamp,Person,Room,Activity,Value

where Value is either ‘on’ (start) or ‘off’ (end).

1. Presence and Entry Detection

  Triggered through ESP32-CAM motion, face recognition assigns a user ID to an entry room. Presence is logged immediately, as outlined in Algorithm 1.
**Algorithm 1** onUserDetected**Require:** 
userId, timestamp**Ensure:** 
An activity log entry is created and the user’s context is updated. 1:room← ENTRY_ROOM 2:**if** userId not in userCtx  **then** 3:   initialize userCtx[userId] 4:**end if** 5:userCtx[userId].currentRoom←room 6:userCtx[userId].currentActivity←null 7:logActivity(timestamp, userId, room, “presence”, “on”)

2. Room Transition Detection

Each NFC scan toggles between two rooms defined for that reader. The transition process is outlined in Algorithm 2.
**Algorithm 2** onRoomScan (with NFC toggle)**Require:** 
userId, tagId, timestamp**Ensure:** 
A room transition is logged and the user’s current room is updated. 1:(roomA, roomB) ← lookupTagRooms(tagId) 2:lastRoom←userCtx[userId].currentRoom 3:**if** lastRoom=roomA **then** 4:   room←roomB 5:**else if** lastRoom=roomB **then** 6:   room←roomA 7:**else** 8:   room←roomA {default on first scan} 9:**end if** 10:userCtx[userId].currentRoom←room 11:logActivity(timestamp, userId, room, “transition”, “on”)

3. Activity Prediction and Automation (with Confirmation)

Upon each room entry, features are built and passed to the EL-HARP model. The predicted activity is confirmed via voice; on affirmation, automation executes and the activity start is logged. This process is summarized in Algorithm 3.
**Algorithm 3** onRoomEnter (with user confirmation)**Require:** 
userId, timestamp**Ensure:** 
An activity is logged and the user’s activity context is updated, or a pending configuration is saved for learning. 1:ctx←userCtx[userId] 2:features← buildFeatures(ctx.currentRoom, ctx.historyVector, ctx.weightVector, timestamp) 3:activity← runModelInference(features) 4:speak(“Are you currently activity?”) 5:reply← getUserResponse() 6:**if** $reply=“{\mathrm{yes}}^{”}$ **then** 7:     executeAutomation(userId, activity) 8:     logActivity(timestamp, userId, ctx.currentRoom, activity, “on”) 9:     ctx.currentActivity.label←activity 10:   ctx.currentActivity.combo← readApplianceStates() 11:   ctx.currentActivity.timestamp←timestamp 12:**else** 13:   ctx.pendingConfig← readApplianceStates() 14:   ctx.timerCount←0 15:**end if**

4. Appliance-Combo Logging and Voice-Prompt Learning

Every 30 s, the system checks whether the current appliance state matches the pending combo. Known combos are logged immediately; unknown ones trigger a voice prompt and update the mapping. This routine is implemented in Algorithm 4.

5. Activity End Detection

  If the appliance combo no longer matches the recorded combo for the active activity for over 10 s, an “off” event is logged, and the activity session is cleared. Algorithm 5 summarizes this process.
**Algorithm 4** checkPersistentCombo and handleCombo**Require:** 
timestamp**Ensure:** 
An activity is logged, and a new activity-appliance combination is learned if not already known. 1:**for all** userId in userCtx **do** 2:    ctx←userCtx[userId] 3:    combo← readApplianceStates() 4:    **if** combo=ctx.pendingConfig **then** 5:         ctx.timerCount+=1 6:    **else** 7:         ctx.pendingConfig←combo 8:         ctx.timerCount←1 9:    **end if** 10:   **if** ctx.timerCount≥30 **then** 11:        **if** isKnownCombo(combo) **then** 12:          label← lookupComboLabel(combo) 13:        **else** 14:          speak(“What are you doing?”) 15:          label← whisperTranscribe(recordAudio()) 16:          updateComboMap(combo, label) 17:        **end if** 18:        logActivity(timestamp, userId, ctx.currentRoom, label, “on”) 19:        ctx.currentActivity←label 20:    **end if** 21:**end for**

**Algorithm 5** detectActivityEnd
**Require:** 


timestamp

**Ensure:** 
An activity-end event is logged and the user’s activity context is cleared. 1:**for all** userId in userCtx **do** 2:    ctx←userCtx[userId] 3:    **if** ctx.currentActivity≠null **then** 4:         currentCombo← readApplianceStates() 5:         **if** currentCombo≠ctx.currentActivity.combo **then** 6:             increment disconnect timer 7:         **else** 8:             reset disconnect timer 9:         **end if** 10:         **if** disconnect timer ≥ 10 s **then** 11:            logActivity(timestamp, userId, ctx.currentRoom, ctx.currentActivity.label, “off”) 12:            ctx.currentActivity←null 13:         **end if** 14:    **end if** 15:
**end for**



Data Storage and Services

- Activity Logs: /home/pi/activity_log.txt, each line ‘Timestamp, Person, Room, Activity, Value’.
- Combo Mappings: /home/pi/combos.json, JSON maps appliance states to activities.
- Voice Files: /tmp/voice.wav and /tmp/voice.wav.txt for temporary audio/transcription.
- Dockerized Services: OpenCV, Home Assistant, Node-RED, Whisper, and inference engine each run in isolated containers on the Pi.

In the remainder of this section, each stage is described in detail:

### 3.3. Data Collection

**Figure 3:**
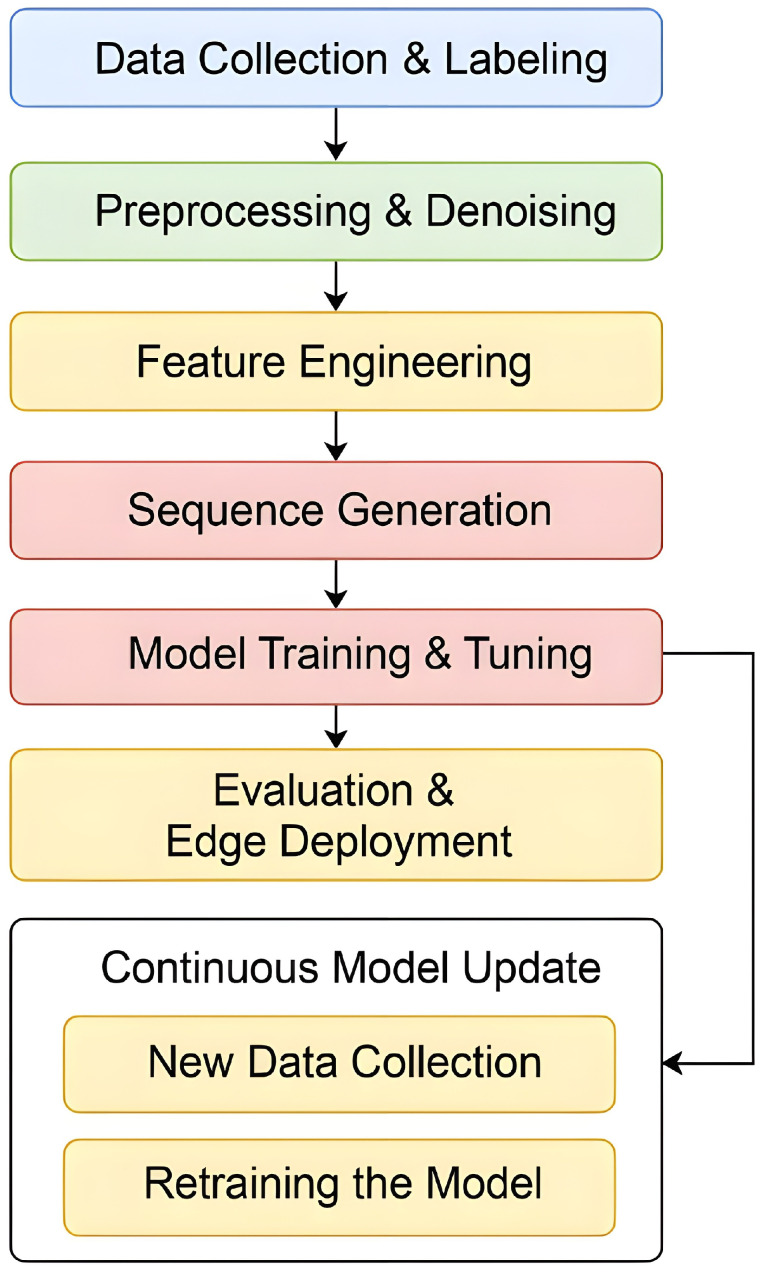
System workflow with continuous retraining loop.

This study’s data collection strategy encompasses two complementary scenarios to support both rigorous evaluation and live personalization. In the proof-of-concept evaluation, experiments are conducted on a curated subset of the publicly available CASAS Smart Home dataset. Sensor events and annotated activities from 21 independent single-resident households—each exhibiting distinct daily routines and environmental contexts—are used to validate the generalizability of the feature engineering and sequence design across diverse living scenarios. These CASAS-trained models are not deployed directly; they serve solely to demonstrate feasibility, with no cross-user transfer in the live system (Box 1 in Figure 3).

In the real-world deployment scenario, EL-HARP operates exclusively on the resident’s own data. The system initializes in a minimal “cold-start” state and continuously collects live sensor events via the Sensing Layer. Each new labeled event—whether resulting from a confirmed model prediction or from a voice-prompted annotation—is appended to the local activity log. At regular, configurable intervals (Box 7 in Figure 1), this freshly accrued data is ingested into the continuous retraining loop, enabling fully local personalization, strict privacy preservation, and adaptation to evolving routines without reliance on any external datasets.

### 3.4. Preprocessing and Denoising

The second step involves cleaning and standardizing the dataset. Given the nature of the CASAS dataset as a long-term, multi-user research resource, the data often contains various forms of noise—such as sensor glitches, overlapping event bursts, and infrequent or mislabeled activity classes. The preprocessing pipeline consists of three main stages:

1. Data Transformation
  - Event Pairing: Each binary sensor logs on/off events. Consecutive on/off pairs for the same sensor define an interval $\left(t_{\mathrm{on}},\;t_{\mathrm{off}}\right)$. Intervals shorter than a threshold (e.g., 2 s) are discarded to reduce spurious noise.
  - Activity Label Assignment: each valid interval is mapped to a pre-annotated CASAS activity label (e.g., Cooking, Sleeping), producing a sequence of timestamped activity intervals.
2. Label Cleaning
  - Noise-Prone Class Removal: Ambiguous or sparsely represented classes (e.g., Other_Activity, Entertain_Guests, ENTER) are removed entirely, as they usually represent sensor noise.
  - Null-Label Filtering: Time steps with $A_{t}=\mathrm{NULL}$ are retained for context, but any sequence whose final label is NULL will be discarded in sequence generation.
3. Class Imbalance Handling:
  - After filtering, if the ratio of majority to minority classes exceeds 10:1, majority-class under-sampling is applied so that no class has more than five times the instances of the smallest class.

It is worth noting that such extensive preprocessing may not be necessary in real-world deployments. A continuously operating system like EL-HARP can enforce stricter data quality at the source (e.g., consistent sensor configuration, real-time logging). Therefore, while this pipeline standardizes historical datasets like CASAS for fair evaluation, future on-device systems may adopt more lightweight validation strategies.

### 3.5. Feature Engineering

At this stage, raw sensor events are transformed into structured numerical representations that capture user behavior across multiple time scales. A combination was extracted of instantaneous features (e.g., current room, time of day), short-term rolling features (e.g., room and activity trends over the past few hours), and long-term historical features (e.g., most frequent past activities across the previous 21 days with decay weighting). This layered design enables the model to learn from both immediate context and recurring daily patterns.

All preprocessing is performed on a per-person, timestamp-sorted dataframe. Categorical variables (Room, Activity) are encoded as integers, and continuous-derived quantities are standardized to zero mean and unit variance within each household. At each timestamp, the following features are computed and grouped into three categories based on their temporal scope:

1. Instantaneous Features (Current Context)
  - Room ID ($R_{t}$): Encoded room identifier at time *t*, where $R_{t}\in \left\{0,\ldots ,R-1\right\}$ and *R* is the number of unique rooms.
  - Time of Day (sinusoidal encoding): The hour of the day $h_{t}\in \left\{0,\ldots ,23\right\}$ is converted to two cyclic features:
    $${\mathrm{time}\_\sin}_{t}=\sin\;\left(2\pi \;\frac{h_{t}}{24}\right),\;{\mathrm{time}\_\cos}_{t}=\cos\;\left(2\pi \;\frac{h_{t}}{24}\right).$$
  - Day of Week ($d_{t}$): An integer representing the weekday, where $d_{t}\in \left\{0,\ldots ,6\right\}$, with 0 corresponding to Monday.
2. Short-Term Rolling Features (Recent Behavior)
  - Hourly Activity Count ($N_{h}\left(t\right)$): Total number of activity events recorded within the hour containing timestamp *t*, defined as follows:
    $$\begin{array}{cc} N_{h}\left(t\right)\;=\;|\{\tau | & \mathrm{hour}\_\mathrm{of}\_\mathrm{day}(\tau )=\mathrm{hour}\_\mathrm{of}\_\mathrm{day}(t),\\ & \mathrm{date}(\tau )=\mathrm{date}(t),\;\mathrm{and}\;A(\tau )\neq \mathrm{null}\_\mathrm{activity}\}|. \end{array}$$
  - Room Entropy ($H_{R}$): A scalar indicating the diversity of room usage by the user, calculated as the Shannon entropy of room visits:
    $$H_{R}\;=\;-\sum_{i=0}^{R-1}p_{i}\;\log p_{i},\;$$
    where $p_{i}$ is the proportion of activity events occurring in room *i* over the entire observation period. This feature is static and computed once per user, but it provides a critical baseline for understanding short-term deviations.
  - Rolling Activity Mean (3 h): The mean of encoded activity values within the 3-h trailing window ending at time *t*:
    $$\mu_{act,3h}\left(t\right)=\frac{1}{|W_{t}^{3h}|}\sum_{\tau \in W_{t}^{3h}}A\left(\tau \right),\;\mathrm{where}\;W_{t}^{3h}=\left\{\;\tau |t-3h\leq \tau \leq t\right\}.$$
  - Rolling Room Mean (6 h): The mean of encoded room ID values within the 6-h trailing window ending at time *t*:
    $$\mu_{room,6h}\left(t\right)=\frac{1}{|W_{t}^{6h}|}\sum_{\tau \in W_{t}^{6h}}R\left(\tau \right),\;\mathrm{where}\;W_{t}^{6h}=\left\{\;\tau |t-6h\leq \tau \leq t\right\}.$$
3. Historical Features (Long-Term Routine)
  To capture long-term periodic behaviors, a 21-day history embedding is constructed:
  - For each day d∈{1,…,21}, the dominant activity label from a 2 h window centered around the same time of day, *d* days prior to *t*, is extracted:
    $$h_{t}\left[d\right]=mode\left\{A(\tau )|\tau \in [t-d\;\mathrm{days}-1h,\;t-d\;\mathrm{days}+1h]\right\}.$$
  - A decay weight, $w_{d}$, is assigned based on the recency and weekday alignment:
  $$w_{d}=\left\{\begin{array}{cc} 1.8, & \mathrm{if}\;d\;\mathrm{is}\;a\;\mathrm{multiple}\;\mathrm{of}\;7\;\mathrm{AND}\;weekday(t-d)=weekday(t),\\ 1.5, & \mathrm{if}\;d\;\mathrm{is}\;a\;\mathrm{multiple}\;\mathrm{of}\;7\;(\mathrm{but}\;\mathrm{not}\;\mathrm{satisfying}\;\mathrm{above}\;\mathrm{condition}),\\ \max\left(0.24,\;0.96-0.12\;d\right), & \mathrm{if}\;weekday(t-d)=weekday(t)\;(\mathrm{but}\;\mathrm{not}\;\mathrm{satisfying}\;\mathrm{above}\;\mathrm{conditions}),\\ \max\left(0.2,\;0.8-0.1\;d\right), & \mathrm{otherwise}. \end{array}\right.$$
  This yields two feature vectors per timestamp:
    $$h_{t}=\left[h_{t}\left[1\right],\ldots ,h_{t}\left[21\right]\right]\in Z^{21},\;w_{t}=\left[w_{1},\ldots ,w_{21}\right]\in R^{21}.$$

### 3.6. Sequence Generation

The fourth step in Figure 3 illustrates how the live sensor stream and logged events are converted into supervised learning samples of fixed length L=30. Each sample consists of the following:

- $X^{\left(i\right)}\in R^{30\times 8}$: the most recent 30 standardized feature vectors;
- $h^{\left(i\right)}\in Z^{D}$: the *D*-day history vector (D=21);
- $w^{\left(i\right)}\in R^{D}$: the corresponding decay weights;
- $y^{\left(i\right)}\in Z$: the activity label at time *i*;

where any sequence with $y^{\left(i\right)}=\mathrm{NULL}$ is discarded.

Sliding Window Extraction

For each timestamp index, i≥L,

$$X^{\left(i\right)}=\left[\begin{array}{c} x_{i-L}\\ x_{i-L+1}\\ \vdots \\ x_{i-1} \end{array}\right]\in R^{30\times 8},\;y^{\left(i\right)}=A_{i}.$$

Feature–History Configurations

  To evaluate the influence of different feature sets on performance, three input configurations are defined:

Configuration 1: Minimal + 7-Day History X uses only instantaneous context (room ID, time_sin, time_cos, weekday), paired with the 7 most recent days in h (and their weights in w).
Configuration 2: Extended + 21-Day History Adds short-term rolling features (hourly activity count, room entropy) to X and extends h and w to 21 days.
Configuration 3: Enhanced + Rolling Statistics + 21-Day History Further includes 3 h and 6 h rolling means in X, retains the full 21-day history, and applies cyclic encodings for time-of-day and weekday.

Data Storage

Each complete training tuple, $\left\{X^{\left(i\right)},\;h^{\left(i\right)},\;w^{\left(i\right)},\;y^{\left(i\right)}\right\}$, is flattened—dropping any columns constant within that household—and appended to an on-device Hierarchical Data Format 5 (HDF5) cache. Encoded class mappings (activity_classes, room_classes) are stored alongside. This cache supports both initial training and the continuous retraining of EL-HARP directly on the Raspberry Pi.

### 3.7. Model Training and Tuning

This stage covers model selection, hyperparameter tuning, and the transition from proof-of-concept to live deployment.

Model Architectures

Three gradient-boosted decision tree (GBDT) ensembles were chosen for activity recognition:

- XGBoost [38]: Histogram-based tree construction, multi-class softmax objective, strong regularization.
- CatBoost [39]: Ordered boosting, native categorical handling, automatic feature combinations.
- LightGBM [40]: Gradient-based one-side sampling (GOSS), exclusive feature bundling (EFB), histogram splitting for low memory.

All models used categorical cross-entropy (multi-class log-loss) with 2% injected label noise to simulate annotation errors. Table 2 summarizes their inference speed, memory footprint, and edge suitability.

Dataset Splitting

For each of the 21 single-resident households, sequences were split into 80% training, 10% validation, and 10% test. Stratification by label was applied when each class had ≥20 samples; otherwise, a random split was used.

Hyperparameter Optimization and Early Stopping

Several hyperparameter configurations were assessed on validation subsets from different users. The setting that consistently achieved the best performance across households was selected and used for all models. Models were trained with early stopping (patience = 100 rounds) on validation log-loss. Common settings: Subsampling and feature-fraction (or Random Subspace Method (RSM)) were both set to 0.8 across all models. Table 3 details the full hyperparameter configurations.

### 3.8. Evaluation and Edge Deployment

The final stage of the EL-HARP framework focuses on model evaluation used for maintaining system personalization over time.

Model performance is assessed per user using three complementary premises:

- Accuracy (Activity Recognition Accuracy): The percentage of correctly predicted activity labels across all test samples.
- Weighted F1-Score: A class-weighted harmonic mean of precision and recall that accounts for label imbalance.
- Confusion Matrix Analysis: A per-class visualization of prediction performance, helping to diagnose misclassification trends between similar or overlapping activities.

All metrics are computed on the test set of each household. Validation scores are calculated using the same metrics and used for early stopping during training. Formal equations and result tables are provided in Section 4.

### 3.9. Continuous Personalization and Edge Adaptation

To ensure long-term adaptability, EL-HARP includes an efficient retraining loop that runs entirely on the deployed edge device.

On-device inference and logging:

The trained LightGBM model and preprocessing pipeline are containerized and deployed on a Raspberry Pi 5. As live sensor events are streamed in, they are transformed into structured feature sequences and passed through the model for real-time prediction. Each instance is then appended to a persistent HDF5 log with timestamp, user ID, room, and predicted activity.

Local incremental retraining:

At scheduled intervals (e.g., weekly), the following update procedure is triggered:

- Newly logged instances are retrieved from the HDF5 cache.
- These samples are appended to the existing training set.
- The LightGBM model is warm-started, extending the existing ensemble with a limited number of trees (e.g., 50).
- Validation metrics are re-evaluated on a held-out portion of recent data.

If performance improves or remains within an acceptable tolerance range, the new model replaces the previous version. Otherwise, the update is discarded to prevent model drift.

## 4. Results and Performance Analysis

The evaluation of XGBoost, CatBoost, and LightGBM is conducted across three different feature setups, as defined in Section 3.7: Configuration 1 (basic feature set with 7-day history), Configuration 2 (an extended feature set with 21-day history), and Configuration 3 (augmented features incorporating rolling statistics over 21 days). The primary performance metrics reported are validation accuracy ($A_{\mathrm{val}}$) and validation F1-score (F_1,val_), along with user-specific test accuracy values for Configuration 3. Additionally, key metrics such as activity recognition accuracy (ARA) and F1-score are used to comprehensively evaluate model performance.

### 4.1. Dataset Transformation and Labeling

Smart home sensor logs are inherently noisy and heterogeneous, containing inconsistent readings, redundant signals, and ambiguous activity labels. In particular, the Single-Resident CASAS dataset [3] includes classes—Other_Activity, Entertain_Guests, and ENTER—that frequently correspond to sensor glitches or uninformative transitions. To focus on meaningful daily routines, these classes were removed from every household’s raw logs, following the filtering methodology of Cook et al. [41].

#### 4.1.1. Filtering Criteria and Class Statistics

After discarding the three noisy classes, each household’s log was reduced to events with well-defined activities (e.g., Sleep, Cooking, Watch_TV, etc.). Table 4 summarizes the effect of this cleaning on a subset of the 21 households.

**Figure 4:**
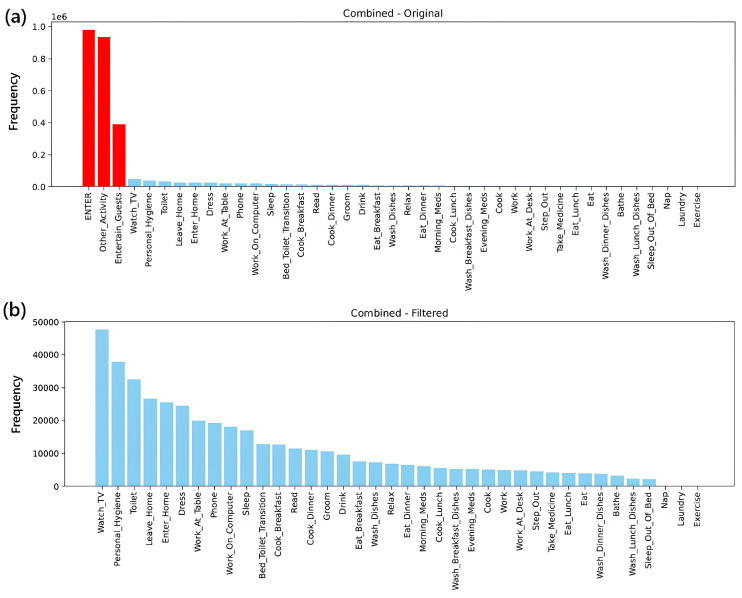
Global distribution of activity classes across 21 households: (**a**) before filtering, showing dominance of noise classes such as ENTER and Other Activity; (**b**) after filtering, illustrating the removal of low-frequency, high-ambiguity labels and the relative preservation of core daily activities.

Figure 4 compares the global class distributions before and after filtering, illustrating the removal of low-frequency, high-ambiguity labels and the relative preservation of core daily activities.

#### 4.1.2. Example: Raw vs. Cleaned Sequence

**Figure 5:**
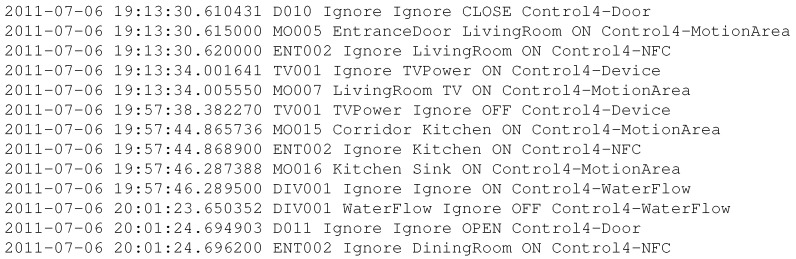
Fabricated raw sensor log with mixed, low-level device events.

**Figure 6:**
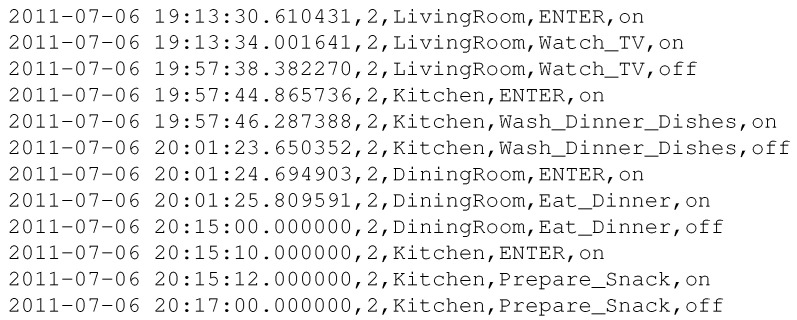
Cleaned, structured sequence ready for feature extraction and modeling.

The following listings provide representative samples of the raw sensor log (before filtering and labeling) and its cleaned, structured counterpart. Figure 5 shows a representative raw sensor log collected from various IoT devices in the smart home. As seen, the log contains low-level, mixed events with redundant and unstructured entries. After preprocessing and labeling, the data is transformed into a clean, structured sequence suitable for feature extraction and modeling, as illustrated in Figure 6.

Raw Sensor Log (Before Processing)

Structured and Labeled Log (After Processing)

#### 4.1.3. Final Class Distribution per Household

**Figure 7:**
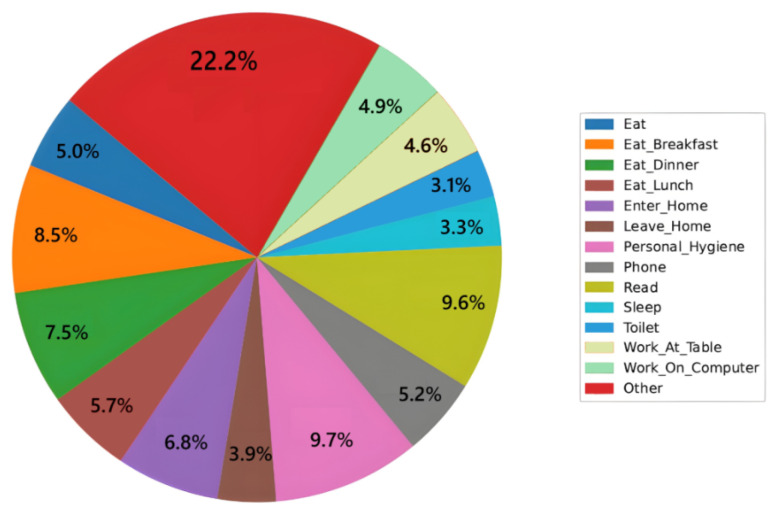
Activity class distribution for a representative household after filtering.

After filtering, the remaining activity classes vary in frequency by household. Figure 7 shows a representative distribution for one home, demonstrating the diversity of daily routines captured post-cleaning.

### 4.2. Evaluation Metrics

Key metrics include the following.

- Activity Recognition Accuracy (ARA): Proportion of correctly predicted activity segments:
  $$\mathrm{ARA}=\frac{\;\mathrm{Correct}\;\mathrm{Activity}\;\mathrm{Segments}}{\;\mathrm{Total}\;\mathrm{Activity}\;\mathrm{Segments}}$$
  In other words, ARA is the overall accuracy of the model on the test set.
- F1-Score: The harmonic mean of precision and recall, particularly useful when activity classes are imbalanced.
  $${F1}_{i}=2\times \frac{{\mathrm{Precision}}_{i}\times {\mathrm{Recall}}_{i}}{{\mathrm{Precision}}_{i}+{\mathrm{Recall}}_{i}}.$$
  The overall (weighted) F1-Score aggregates ${F1}_{i}$ across all *K* classes by weighting each class’s F1 by its support:
  $${F1}_{w}=\sum_{i=1}^{K}w_{i}\cdot {F1}_{i},\;w_{i}=\frac{n_{i}}{\sum_{j=1}^{K}n_{j}}.$$

### 4.3. Overall Validation Performance

Table 5 displays the average ± standard deviation of both $A_{\mathrm{val}}$ and F_1,val_ across 21 users.

A consistent upward trend is observed from Configuration 1 to Configuration 3 across all models, with Configuration 3 yielding a 17–20 percentage point improvement in validation accuracy. LightGBM demonstrates the strongest overall performance in Configuration 3 for both metrics.

### 4.4. User-Level Test Accuracy for Configuration 3

The performance of each model on test data is further broken down across individual users under Configuration 3, as summarized in Table 6.

Among all three models, LightGBM records the highest average test accuracy of 91.5% and exhibits the lowest degree of variation across users.

### 4.5. Model Generalization Behavior

The generalization gap, denoted as $\Delta A=A_{\mathrm{train}}-A_{\mathrm{val}}$, reflects how well the model performance transfers from training to unseen data. Table 7 presents the mean and standard deviation of ΔA for all feature configurations.

All models achieve substantial reductions in generalization gap under Configuration 3, with values falling below 0.04, reflecting improved robustness and generalization to unseen data.

### 4.6. Results Interpretation

Evaluation verifies that EL-HARP’s multi-scale architectural choice—a combination of historical context, historical rolling statistics, and long-term history embeddings—the lightweight LightGBM achieves state-of-the-art performance (91.5% mean test precision). The performance matches, or surpasses even, that of deeper neural methods without compromising interpretability or efficiency. In-device trials under 100 ms with a Raspberry Pi 5 exhibit low-memory-footprint capabilities to confirm end-to-end in-device capabilities with stringent in-device data privacy. The dynamic retraining capability also retains sustained accuracy with dynamic user processes, concluding the limitation of static models in the absence of human intervention.

End-to-end containerized deployment—bundling preprocessing, feature extraction, inferencing from the model, and control logic all into modular services—enables fast deployment, easy updating, and easy interoperation with heterogeneous smart home platforms.

Collectively, all of these experiments demonstrate that EL-HARP provides a versatile, secure, and scalable home automation environment with a forward-looking orientation—with applications for energy use, elderly care, and context-dependent comfort enhancement.

## 5. Comparative Analysis

The optimal configuration—utilizing LightGBM combined with Configuration 3 features—demonstrates a compelling performance when compared against other distinguished smart home Human Activity Recognition (HAR) studies, as summarized in Table 8.

Relative to other HAR techniques, EL-HARP is distinctive in its proposed LightGBM model, where the best chosen Configuration 3 features achieve the 91.5% mean test accuracy.

- Ali et al. [20] achieved 86.03% (F1) using an IoT-Edge framework that focused on anomaly detection for monitoring the elderly. With more powerful general-purpose recognition capacity, EL-HARP outperforms this.
- Feng et al. [21] aimed at medical sensor fusion in an intensive care unit setting, achieving 85.0% F1, showing the domain gap where EL-HARP performs better with higher accuracies in smart home settings.
- Chen et al. [22] achieved 85.63% (F1) through the use of self-supervised learning and attention in a smart home environment. With a more straightforward architecture and superior interpretability, EL-HARP performs better than this.
- Srivatsa and Plötz [23] developed a GNN-based HAR model that produced an F1 score of 88.7%, while EL-HARP’s lighter, non-deep LightGBM model produced even better accuracy.
- Fiori et al. [24] demonstrated GNN-XAR, an explainable GNN with an accuracy of 86.5% that was trained on CASAS data. With less computational overhead, EL-HARP performs five percentage points better than this.
- Zhou et al. [25] proposed TinyHAR, which achieved an accuracy of 89.0% in edge environments. While outperforming TinyHAR, EL-HARP maintains edge efficiency.
- Khan et al. [26] developed a CNN-LSTM hybrid model for HAR, which achieved an accuracy of 90.89%. With a gain of 0.61%, EL-HARP performs better than it while avoiding the complexity of deep learning.
- Dao et al. [28] achieved a high accuracy of 97.35% on the UCI HAR dataset with a real-time system for firefighter activity recognition. However, this approach relies on specific wearable sensors and a single modality, making it less generalizable and not directly comparable to HAR in a multi-sensor ambient smart home environment.
- Li et al. [30] demonstrated a state-of-the-art accuracy of 96.57% on the CASAS dataset using a complex deep learning model. While their model achieves a higher raw accuracy, its computational cost and complexity are significantly greater. In contrast, EL-HARP demonstrates that with carefully engineered features, a lightweight, non-deep model can achieve highly competitive performance, making it a more practical solution for resource-constrained edge devices.

These comparisons illustrate the efficacy of the proposed approach. Key takeaways include the following.

- Comprehensive Feature Engineering: even with non-deep models, performance is improved by incorporating temporal history, contextual windows, and rolling statistics.
- Gradient Boosting Ensembles: LightGBM is perfect for edge deployment since it achieves competitive or better accuracy than intricate deep architectures while using a lot less memory and inference time.
- Personalized Modeling: due to their ability to capture distinct activity patterns and routines, user-specific models customized for each household routinely outperform general-purpose models.

All other conditions being equal, EL-HARP utilizes the interplay of efficient modeling and robust feature building. This paper shows that less complex tree models could possibly dominate the best current deep learning architectures without compromising deployability on small smart home devices—with the use of densely-built features and special training. Smart home automation could be spread and expanded in practice as an outcome of this compromise between performance and efficiency.

## 6. Implications for Smart Home Automation

The empirical findings presented in Section 4 highlight several important directions for practical, personalized smart home deployment:

- Scalable Per-User Personalization: with an average test accuracy of over 90% for all households (Table 6), the findings validate that household-specific, lightweight models can facilitate reliable, context-aware automation.
- Feature Design over Architectural Complexity: the performance gains from Configuration 1 to Configuration 3 imply that meticulously designed historical and temporal features have a greater influence than more complex models.
- Resilience to Behavior Variability: the reduced generalization gap in Configuration 3 (Table 7) demonstrates that long-term behavior modeling can accommodate irregular routines and improve robustness, although short, transitional events remain challenging.
- Suitability for Edge Deployment: LightGBM supports local computation and user privacy, making it appropriate for real-time activity recognition on embedded systems like Raspberry Pi due to its small memory footprint and fast inference speed.

These findings lend support to the design approach that prioritizes effective ensemble techniques, along with strong, domain-informed feature engineering to enable adaptive, privacy-preserving automation in actual smart homes.

## 7. Conclusions

Here, an edge-computing-enabled, scalable, and holistic architecture, called **EL-HARP**, where HARP refers to a Human Activity Recognition framework with Advanced Robotics capabilities—Personalized sMART—has been introduced. EL-HARP offers real-time edge computing automation with lightweight gradient-boosted models in edge computing environments.

After thorough testing with the CASAS dataset, EL-HARP, under LightGBM runtime with Feature Configuration 3, attained an average test precision of 91.5%. Some of the current best algorithms fall short of this figure. These experiments attest that decision tree boosting with gradients works effectively with deeper learning algorithms with heavily contextual and temporal features but remains computationally fast in equilibrium with edge systems. In order to deal with some of the major challenges in smart home automation, such as privacy and personalization, generalization across users, low-latency edge inferencing, and incremental adaptation, EL-HARP has also been implemented over fully functional prototypes with a Raspberry Pi 5, ESP32-CAM, and Tuya-compatible sensors.

## Figures and Tables

**Table 1:** Summary of representative machine learning techniques for smart home HAR.

Study (Year)	Dataset(s)	Method	Key Advantage	Reported Performance
Ali et al. (2025) [20]	CASAS TM029	IoT Edge Framework	Real-time elderly monitoring on edge devices	86.03% (F1)
Feng et al. (2024) [21]	CASAS Aruba/Milan	Masked Modeling (Transformer)	Robust to missing sensor events	85.0% (Aruba F1), 64.0% (Milan F1)
Chen et al. (2024) [22]	CASAS Aruba/Milan	SSL + Self-Attention	Reduces reliance on labeled data	85.63% (F1)
Srivatsa & Plötz (2024) [23]	CASAS (Multiple)	Graph Neural Networks	Captures spatial-temporal structure	78.3–88.7% (F1)
Fiori et al. (2025) [24]	CASAS Milan/Aruba	GNN-XAR	Interpretable GNN with attention graphs	86.5% (Accuracy)
Zhou et al. (2022) [25]	Multiple HAR Datasets	TinyHAR (Edge DL)	Lightweight design for microcontrollers	89.0% (Accuracy)
Khan et al. (2022) [26]	CASAS Dataset	CNN + Bi-LSTM Hybrid	Strong spatio-temporal feature modeling	89.0% (Accuracy)
Dao et al. (2025) [28]	UCI HAR	RFAR (Wearable System)	Real-time, high accuracy on wearable data	97.35% (Accuracy)
Li et al. (2023) [30]	CASAS	Deep Learning (HENN-MSD)	High accuracy on multi-environment data	96.57% (Accuracy)

**Table 2:** Edge deployability characteristics of selected models.

Model	Inference Speed	Memory Footprint	Edge Suitability
XGBoost	Medium	Medium	High–Moderate (requires tuning)
CatBoost	Fast	Low	High (native categorical handling)
LightGBM	Very Fast	Very Low	Very High (optimized for embedded deployment)

**Table 3:** Hyperparameter settings for XGBoost, CatBoost, and LightGBM.

Parameter	XGBoost	CatBoost	LightGBM
Learning rate	0.10	0.05	0.01
Depth/Leaves	6	6	15
Number of estimators/iterations	300	1000	1000
Subsample fraction	0.80	0.80	0.80
Feature-fraction/RSM	0.80	0.80	0.80
Regularization (L1/L2)	2.0/2.0	0.0/2.0	0.0/0.10
Minimum child weight/samples	5	10	50
Early stopping rounds	100	100	100

**Table 4:** Cleaning summary per household: samples before and after removal of noisy classes.

Household	Original	Cleaned	Removed
user 2	104,856	15,672	89,184
user 3	42,048	6522	35,526
user 4	121,865	28,276	93,589
user 5	46,981	9484	37,497
user 6	90,017	14,024	75,993
…	…	…	…
Total	2,731,903	428,914	2,302,989

**Table 5:** Mean and standard deviation of validation accuracy and F1-score.

	Configuration 1		Configuration 2		Configuration 3	
Model	$A_{\mathrm{val}}$	F_1,val_	$A_{\mathrm{val}}$	F_1,val_	$A_{\mathrm{val}}$	F_1,val_
XGBoost	0.72 ± 0.05	0.73 ± 0.04	0.77 ± 0.04	0.76 ± 0.04	0.89 ± 0.03	0.89 ± 0.03
CatBoost	0.65 ± 0.06	0.64 ± 0.05	0.83 ± 0.03	0.82 ± 0.03	0.91 ± 0.02	0.91 ± 0.02
LightGBM	0.72 ± 0.05	0.72 ± 0.05	0.80 ± 0.04	0.79 ± 0.04	0.92 ± 0.02	0.92 ± 0.02

**Table 6:** User-level test accuracy statistics for Configuration 3.

Model	$\min(A_{\mathrm{test}})$	$\max(A_{\mathrm{test}})$	$\mathrm{mean}(A_{\mathrm{test}})$
XGBoost	0.764	0.937	0.887 ± 0.04
CatBoost	0.821	0.956	0.905 ± 0.03
LightGBM	0.821	0.961	0.915 ± 0.03

**Table 7:** Average generalization gap (ΔA) for each configuration.

Model/Configuration	Configuration 1	Configuration 2	Configuration 3
XGBoost	0.24 ± 0.05	0.10 ± 0.03	0.04 ± 0.02
CatBoost	0.20 ± 0.06	0.04 ± 0.02	0.03 ± 0.02
LightGBM	0.22 ± 0.06	0.14 ± 0.04	0.03 ± 0.02

**Table 8:** Comparison with prior smart home HAR approaches.

Study	Approach	Accuracy/F1 Score
Ali et al. (2025) [20]	IoT and Edge Intelligence Framework using anomaly detection	86.03% (F1)
Feng et al. (2024) [21]	ICU Command Center with medical sensor fusion	85.0% (F1)
Chen et al. (2024) [22]	Self-Supervised Learning and Self-Attention in smart homes	85.63% (F1)
Srivatsa & Plötz (2024) [23]	Graph-based HAR using multimodal sensor GNNs	88.7% (F1)
Fiori et al. (2025) [24]	Explainable GNN-XAR on CASAS datasets	86.5% (Accuracy)
Zhou et al. (2022) [25]	TinyHAR: Lightweight deep learning for edge devices	89.0% (Accuracy)
Khan et al. (2022) [26]	Hybrid CNN-LSTM for time-sequential HAR	90.89% (Accuracy)
Dao et al. (2025) [28]	RFAR (Wearable System) for firefighter activities	97.35% (Accuracy)
Li et al. (2023) [30]	Deep Learning (HENN-MSD) for multi-environment data	96.57% (Accuracy)
This work (2025)	LightGBM + Configuration 3 Features (EL-HARP)	91.5% (Accuracy)

## Data Availability

No new data were created or analyzed in this study. Data sharing is not applicable to this article.
